# *Phytophthora* × *cambivora* as a Major Factor Inciting the Decline of European Beech in a Stand within the Southernmost Limit of Its Natural Range in Europe

**DOI:** 10.3390/jof8090973

**Published:** 2022-09-18

**Authors:** Mario Riolo, Francesco Aloi, Sebastiano Conti Taguali, Antonella Pane, Massimo Franco, Santa Olga Cacciola

**Affiliations:** 1 Department of Agriculture, Food and Environment, University of Catania, 95123 Catania, Italy; 2 Department of Agricultural Science, Mediterranean University of Reggio Calabria, 89122 Reggio Calabria, Italy

**Keywords:** *Fagus sylvatica*, *Phytophthora gonapodyides*, root rot, bleeding cankers, nature reserve, leaf-baiting, mating type, ITS, *Cox* I, phylogenetic analysis

## Abstract

The objective of this study was to investigate the role of the oomycete *Phytophthora* *× cambivora* in the decline affecting European beech (*Fagus sylvatica*) in the Nebrodi Regional Park (Sicily, southern Italy). In a survey of a beech forest stand in the heart of the park, *Phytophthora* *× cambivora* was the sole *Phytophthora* species recovered from the rhizosphere soil and fine roots of trees. Both A1 and A2 mating type isolates were found. Direct isolation from the stem bark of trees showing severe decline symptoms and bleeding stem cankers yielded exclusively *P. gonapodyides*, usually considered as an opportunistic pathogen. The mean inoculum density of *P.* *× cambivora* in the rhizosphere soil, as determined using the soil dilution plating method and expressed in terms of colony forming units (cfus) per gm of soil, the isolation frequency using leaf baiting, and the percentage of infected fibrous roots from 20 randomly selected beech trees with severe decline symptoms (50 to 100 foliage transparency classes) were 31.7 cfus, 80%, and 48.6%, respectively. These were significantly higher than the corresponding mean values of 20 asymptomatic or slightly declining trees, suggesting *P.* *× cambivora* is a major factor responsible for the decline in the surveyed stand.

## 1. Introduction

**Figure 1:**
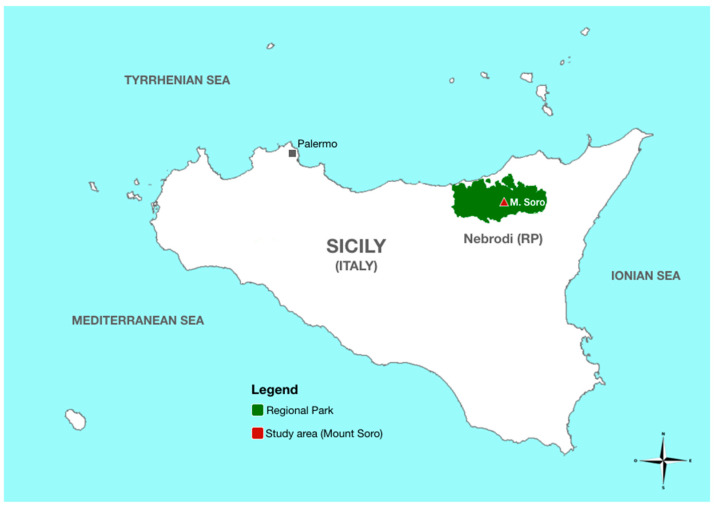
Geophysical map of Sicily with detail of the study area.

**Figure 2:**
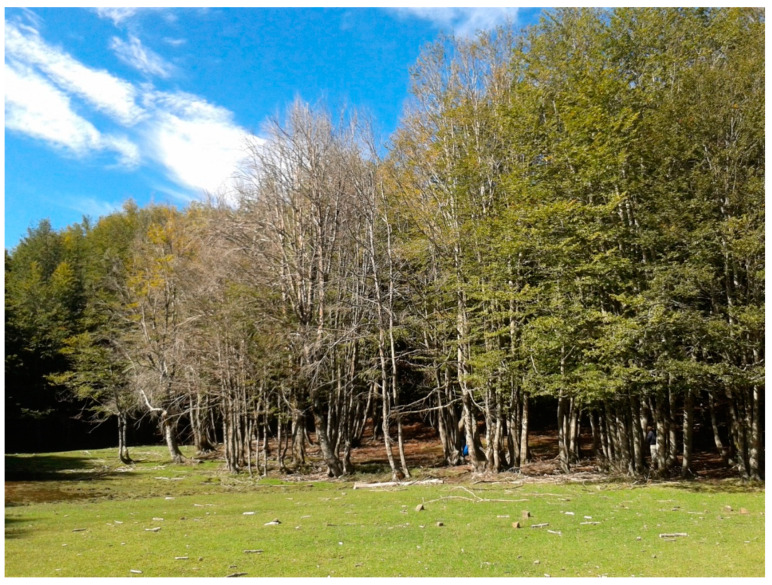
A group of European beech (*Fagus sylvatica*) trees with severe symptoms of decline on the shore of a seasonal water basin in the Nebrodi Regional Park (Sicily, southern Italy).

The Nebrodi Regional Park (RP), spanning nearly 86,000 ha, is the largest protected natural area (PNA) in Sicily (southern Italy) (Figure 1). According to the classification proposed by IUCN (International Union for the Conservation of Nature, Gland, Switzerland), this park is included in the management category IV, encompassing PNAs aimed at protecting particular species or habitats [1]. The Nebrodi RP comprises diverse altitudinal zones ranging from coastal areas of the Tyrrhenian Sea, a few meters above sea level (a.s.l.), to Mount Soro (1847 m a.s.l.), which is the highest peak of the Nebrodi mountain range. At over 1200 m a.s.l., the vegetation of the Nebrodi RP is of the supra-Mediterranean type and includes forest stands dominated by European beech (*Fagus sylvatica*, family *Fagaceae*) (Figure 2), which are considered the southernmost limit of the natural distribution range of beech in Europe [2]. During the last decades beech stands in the Nebrodi RP are experiencing a severe decline and dieback of uncertain etiology. A similar decline of European beech in central Europe was imputed to the combined effect of climatic extremes and infections by *Phytophthora* species [3]. The hybrid species *Phytophthora* × *cambivora* (formerly *P. cambivora*) and other soil-borne *Phytophthora* species were reported to be associated with decline and bleeding stem cankers of European beech in Europe and the USA [4,5,6,7,8,9]. A recent study provided circumstantial evidence the infections by *Phytophthora* species are a major factor inciting the decline of European beech stands in Austria [10]. *Phytophthora* × *cambivora* was the prevalent *Phytophthora* species recovered from both bark cankers and rhizosphere soil of declining beech trees in this country. Moreover, it was found that the geological substrate influenced the distribution of this pathogen, which was exclusively found on geological substrates forming acidic soils with high contents of clay and sand, with a tendency for temporary waterlogging [10]. In a previous survey of PNAs in Sicily, *P.* × *cambivora* was found to be widespread in beech and oak stands of the Nebrodi RP and was the *Phytophthora* species recovered most frequently from the rhizosphere soil of declining beech and oak trees [11]. Therefore, it was hypothesized that the interaction between root infections by this *Phytophthora* species and climate change is the main factor responsible for the decline of European beech in the Nebrodi RP [12]. In Italy, and more generally in central and southeastern Europe, *P.* × *cambivora* is also the prevalent *Phytophthora* species responsible for ink disease of chestnut (*Castanea sativa*), a species of the same family as beech. In recent surveys, it was recovered, although occasionally, from the soil of declining forests of cork oak (*Quercus suber*) in Sardinia and from the rhizosphere soil of diverse species of oak and holly (*Ilex aquifolium*) in the Madonie RP, in Sicily [11,13,14,15,16]. Despite several lines of circumstantial evidence suggesting a major role of *P.* × *cambivora* as a stressor inciting the decline of beech stands of the Nebrodi RP, no conclusive evidence of this has been provided. The objective of this study was to verify the hypothesis that infections by *P.* × *cambivora* are responsible for the decline of beech trees in the Nebrodi RP. To this aim, the relationship between the severity of decline, the presence of *P.* × *cambivora* in the soil, the incidence of fine root infections, and the quantity of inoculum of this oomycete in the rhizosphere soil of beech trees in a representative beech stand in the heart of the RP was investigated.

## 2. Materials and Methods

**Figure 3:**
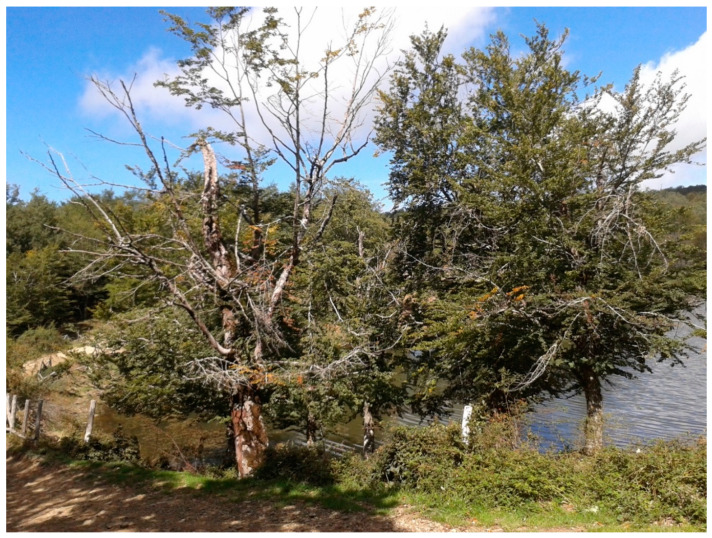
Severely declining trees of European beech (*Fagus sylvatica*) on the shore of Maulazzo Lake in the Nebrodi Regional Park (Sicily, southern Italy).

Sampling activities were carried out from the last week of May to the first week of June 2020 in an almost pure, mature (>60 years old) beech forest stand of Nebrodi RP. A circular area (about 28 km^2^), with a diameter of around 6 km, was ideally delimited around Mount Soro (37°55′53′′ N, 14°41′39′′ E), in the municipality of Cesarò, province of Messina (ME), Sicily, southern Italy. It also encompassed the high altitude Biviere Lake (37°57′11′′ N, 14°42′52′′ E), in the same municipality, and the high altitude Maulazzo Lake (37°56′31′′ N, 14°40′18′′ E), in the municipality of Alcara li Fusi (ME). The geological nature of the soil was marly claystone. Individual scattered trees or groups of trees showed severe decline symptoms. Overall, samples were taken from 40 beech trees of the same age and size (around 60–70 years old and 20–25 m in height, respectively): 20 trees were apparently healthy or with mild symptoms of canopy thinning, while 20 showed severe symptoms of decline, including canopy thinning, twig and branch dieback, leaf yellowing, extensive dieback of the whole crown, and, in a few cases (five trees), bleeding stem cankers (Figure 3). The trees were separated into two groups based on the severity of decline symptoms, which was rated visually according to the classification of the crown condition (defoliation and foliage transparency) by Schomacher et al. and Eichhorn et al. [17,18]. Trees within the 0 to 25 foliage transparency classes were considered healthy, while trees within the 50 to 100 foliage transparency classes were included in the group of declining trees. The 40 trees were randomly selected across the study area. However, all the 40 selected trees were at least 150 m apart from each other to exclude the clustering of data and the small-scale effect of the sampling site. Sampled trees were georeferenced using a portable GPS tracker (GPS AT-17; Autoseeker Electronics Co. Ltd., Shenzhen, China), to avoid sampling bias and sampling overlapping. 

### 2.1. Sampling, Isolation, and Quantitative Determination of Phytophthora

Rhizosphere soil samples, including fine roots, were collected under the canopy of the 40 selected beech trees after the removal of the upper organic soil layer (ca. 5–10 cm). Soil sampling and isolation were performed according to [11,19,20]: four soil cores were collected from each tree, 50–150 cm away from the stem base, and were bulked together (about 2 L). Two subsamples of about 500 mL were used for baiting tests that were performed in a walk-in growth chamber with 12 h natural day light at 20 °C. Each soil sample was placed in a plastic container and flooded with distilled water. Young leaves of carob-tree (*Ceratonia siliqua*) and oak (*Quercus* spp.) floated over flooded soil were used as baits. Organic debris floating on the water surface was removed prior to placing the bait leaflets. After 24–48 h incubation, leaves developing necrotic areas were checked under the light microscope at ×80 magnification for *Phytophthora* sporangia, and then leaf pieces (size 2 mm) from necrotic lesions were plated in Petri dishes onto selective PARPNH agar medium, which consisted of 100 mL V8 juice (Campbell Grocery Products Ltd., Ashford, UK), 15 g agar, 3 g CaCO_3_, 200 mg ampicillin, 10 mg rifampicin, 25 mg pentachloronitrobenzene (PCNB), 50 mg nystatin, 50 mg hymexazol, and 1 L of deionized water. Petri dishes were incubated at 20 °C in the dark. Outgrowing *Phytophthora* hyphae were transferred onto V8 juice agar (V8A) under the stereomicroscope.

For direct isolation of *Phytophthora* from roots, a soil subsample of about 1 L was sieved to separate fine roots. Roots were randomly selected, washed with tap water, rinsed with distilled water, blotted dry, and cut into small segments (5–6 mm). Root segments were plated in Petri dishes onto selective PARPNH agar medium (8 segments per Petri dish and 10 Petri dishes with each soil sample). Petri dishes were incubated at 20 °C in the dark. *Phytophthora* colonies were subcultured on V8 juice agar (V8A). 

A dilution-plate technique was used for the quantitative determination of *Phytophthora* inoculum in the soil. An aliquot of 100 mg from each original soil sample was suspended in 1 L of sterile distilled water (s.d.w.). The suspension was mixed on a stirrer, and, while it was still stirring, it was pipetted into empty Petri dishes (1 mL per Petri dish and 10 dishes for each sample). Selective PARPNH agar medium was poured into the dishes at super fusion temperature (around 43 °C). The dishes were gently rotated to mix the soil suspension uniformly with the selective agar medium, and, after solidification of the medium, they were incubated at 20 °C in the dark. Colonies of *Phytophthora* were counted after three and six days of incubation. Randomly selected colonies were subcultured on PARPNH agar medium and subsequently on V8A for identification. Results were expressed in terms of colony forming units (cfu) g-1 of soil and were corrected by calculating the oven-dry weight of two subsamples (20 mg each) for each original soil sample analyzed.

For direct isolation from bleeding stem cankers, bark pieces (around 10–15 cm in length), including phloem and cambium, were excised from active advancing front of cankers, wrapped tightly with wetted paper, placed in a plastic bag, and taken to the laboratory. Before isolation, they were rinsed with tap water and blotted dry with sterile filter paper. Small bark pieces (2 mm) were picked up with a sterile scalpel and plated in Petri dishes onto selective PARPNH agar medium. Petri dishes were incubated at 20 °C in the dark. Outgrowing *Phytophthora* hyphae were subcultured on V8A. 

All the *Phytophthora* isolates obtained from baiting or direct isolation from soil, roots, and stem bark were maintained on V8A in the dark at a temperature of 6 °C.

### 2.2. Morphological Identification of Isolates

Morphological features and colony morphology of isolates were determined on colonies grown on potato dextrose agar (PDA; Oxoid Ltd., Basingstoke, UK) and V8A at 24–26 °C in the dark, according to standard procedures as described by Riolo et al. [19]. Cardinal temperatures for radial growth were determined by growing the isolates on PDA in Petri dishes (9 cm diam.) and incubating the dishes at 5, 10, 15, 20, 25, 30 and 35 °C (all ± 0.5 °C), in the dark, with four replicates per each isolate and temperature value. Sporangia production was stimulated with the method described by Santilli et al. [21]. Fragments of 2 mm were cut from the growing edge of 7-day-old cultures grown in Petri dishes (15 mm diam.) on V8A at 27 °C in the dark, and they were placed in a 5 cm diameter Petri dish and flooded with non-sterile soil extract water (200 g soil suspended in 1 L of deionized water for 24 h at room temperature and then filtered). After incubation, at 27 °C in the dark for 24–48 h, dimensions and morphological features of 50 mature sporangia of each isolate were determined at ×400 magnification.

### 2.3. Molecular Identification of Isolates

The DNA of the pure cultures of isolates obtained from soil, roots, and bark was extracted by using PowerPlant Pro DNA isolation Kit (MO BIO Laboratories, Inc., Carlsbad, CA, USA), in accordance with the protocol of the manufacturer. The DNA was preserved at −20 °C. The molecular identification of *Phytophthora* species was performed by the analysis of internal transcribed spacer (ITS) of ribosomal DNA (rDNA) and the mitochondrially encoded cytochrome oxidase subunit I (*Cox* I) regions. DNA for ITS sequencing was amplified using forward primer ITS6 and reverse primer ITS4 [22,23]. The PCR amplification mix and thermocycler conditions were in accordance with Cooke et al. [22]. All PCRs were carried out in a 25 µL reaction mix containing PCR buffer (1×), dNTP mix (0.2 mM), MgCl_2_ (1.5 mM), forward and reverse primers (0.5 mM each), Taq DNA Polymerase (1 U), and 100 ng of DNA. The thermocycler conditions were as follows: 94 °C for 3 min, followed by 35 cycles of 94 °C for 30 s, 55 °C for 30 s, 72 °C for 30 s, and then 72 °C for 10 min. *Cox* I was amplified for sequencing using the primers COXF4N and COXR4N [24]. Amplification reactions were conducted in final concentrations of genomic DNA at 1 to 3 ng µL^−1^, 200 μM dNTP (2.0 mM), 0.4 μM ITS6 Phy-8b and Phy-10b primers, 2% glycerol, 3 mM MgCl_2_, 1× buffer, and AmpliTaq polymerase (Applied Biosystems) at 0.05 units of µL^−1^. PCR conditions were as follows: 1 cycle of denaturation at 95°C for 3 min; 35 cycles of 1 min at 95 °C, 1 min of annealing at 65.5 °C, and 1 min of extension at 72 °C; followed by 1 extension cycle at 72 °C for 5 min. Amplicons were detected in 1% agarose gel and sequenced in both directions by an external service (Amsterdam, The Netherlands). Derived sequences were analyzed using FinchTV v.1.4.0 (Geospiza Inc., Seattle, WA, USA) [25]. For species identification, blast searches [26] in the Phytophthora Database [27], GenBank [28], and in a local database containing sequences of ex-type or key isolates from published studies were performed. Isolates were assigned to a species, when their sequences were 99–100% identical to a reference isolate.

### 2.4. Mating Type of Isolates

The mating type of *P.* *×* *cambivora* isolates was determined in dual cultures with A1 and A2 tester strains. The test was performed in Petri dishes (diameter 90 mm) on V8A. The dishes were incubated at 25 °C in the dark and, after 15 d of incubation, were examined under the microscope for the formation of gametangia. Preliminary, 20 randomly selected *P.* × *cambivora* isolates obtained from baits were paired, in all possible combinations, with four tester strains of *P. nicotianae* from the collection of the Molecular Plant Pathology laboratory (University of Catania), C301 (A2 type), Pandorea 2c (A2 type), Ferrara R11 (A1 type), and Serravalle 1 (A1 type), as characterized in previous studies [29,30]. Two *P.* × *cambivora* isolates of A1 mating type and two of A2 mating type were randomly selected among the 10 isolates tested preliminarily and were used as tester strains in subsequent tests to determine the mating type of randomly selected *P.* × *cambivora* isolates recovered from leaf baits, roots, and soil. Indirect tests for sexual compatibility type were carried out using 0.2 μm polycarbonate membrane, as described by Ko (1978) [31]. Isolates were grown on 15 mL HSA (amended with 30 mg β-sitosterol L^−1^) in 9 cm diameter plastic Petri dishes for 3 days at 20 °C in the dark. All isolates were paired with themselves in all possible combinations and with A1 (Correa8) and A2 (STA24) mating types of *P. nicotianae* [30].

### 2.5. Statistical Analysis of Results

Data from individual scattered trees or groups of trees were analyzed using RStudio v.1.2.5. To perform correlation analysis, which measures a linear dependence between two variables, Pearson correlation test was performed. Correlation is significant at the 0.01 level. Log and angular transformations were applied to the values of ID and percentage of infected fine roots, respectively, to obtain a normal distribution. Analysis of variance revealed no significant differences in inoculum density and infected roots among all trees analyzed (*p* value > 0.01), except between inoculum density and infected roots of trees with mild symptoms (*p* = 0.001027). K-means cluster analysis was performed on standardized values of the three variables (proportion of infected fibrous roots, foliage transparency, and inoculum density), using the packages cluster of RStudio v.1.2.5 [32].

## 3. Results

**Figure 4:**
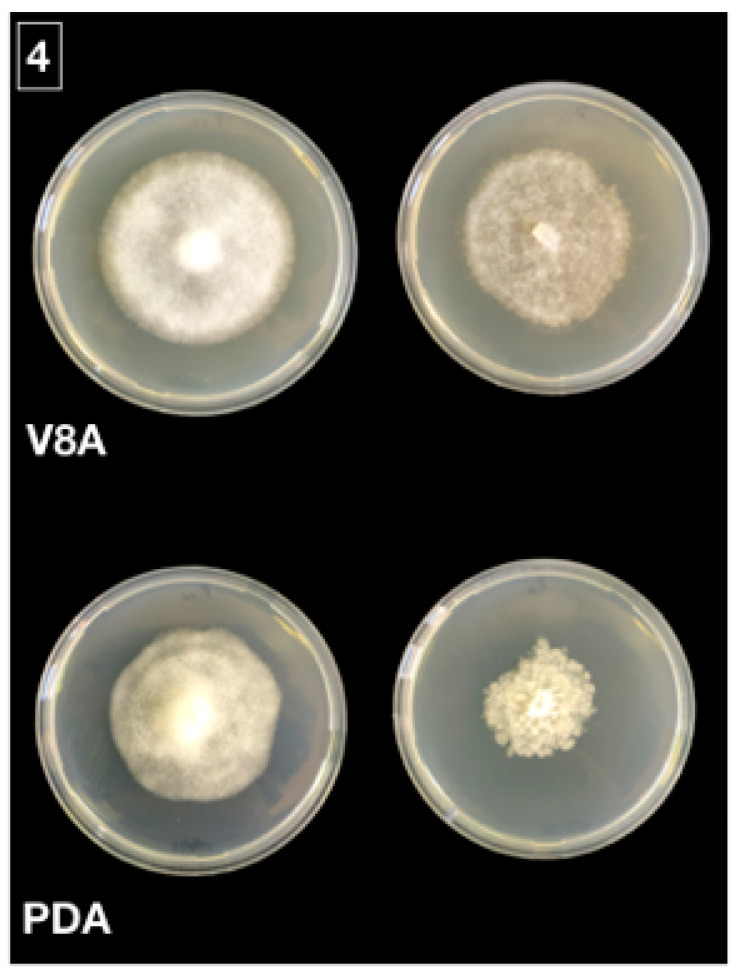
Colony morphologies of *Phytophthora* *× cambivora* (upper row and bottom row to the left; isolate MSK1A) and *P. gonapodyides* (upper row and bottom row to the right; isolate MSC1A) after 4 days growth at 20 °C in the dark on V8A and PDA.

**Figure 5:**
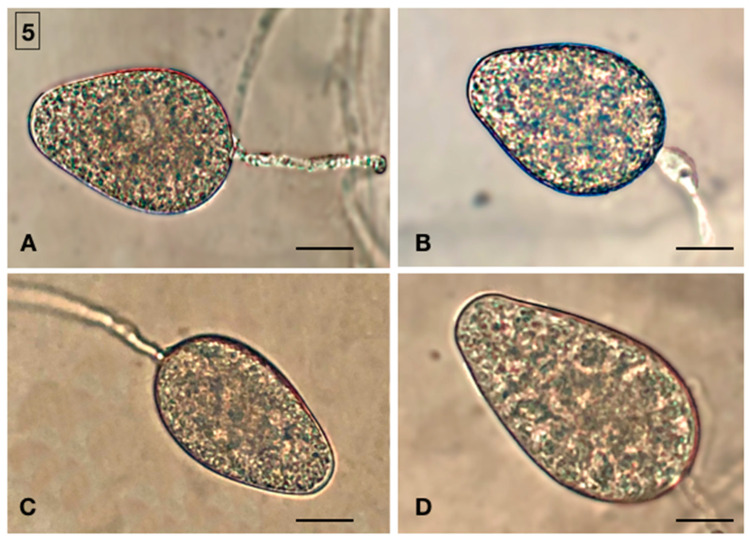
Sporangia of *Phytophthora* *× cambivora* and *P. gonapodyides*. (**A**,**B**) Non-papillate, persistent, and ovoid sporangia of *P.* *× cambivora* (**C**,**D**). Non-papillate, persistent, and ellipsoid sporangia of *P. gonapodyides*. Scale bar: 25 μm.

Isolations from leaf baits, roots, and rhizosphere soil of symptomatic and asymptomatic beech trees sampled in the forest stand of Mount Soro in the Nebrodi RP yielded *Phytophthora* isolates all with the same colony morphology. In total, 30 (10 from leaf baits, 10 from fine roots and 10 from soil) randomly selected, single-hypha tip isolates were characterized. The isolates formed uniform, woolly colonies with a coralloid mycelium on both V8 agar (V8A) and potato dextrose agar (PDA) (Figure 4). Minimum and maximum temperature were below 5 °C and above 35 °C, respectively, with an optimum at 25 °C. Plugs from cultures grown on V8A flooded with non-sterile soil extract produced ovoid, elongated, less frequently ellipsoid, obpyriform, or limoniform, non papillate to semipapillate, persistent, and internally proliferating sporangia (Figure 5). Sporangium dimensions averaged 65 ± 11 × 40 ± 6 μm. Chlamydospores were absent. All isolates were heterothallic and formed antheridia, oogonia, and oospores only, when paired with reference *P. nicotianae* isolates of the A1 and A2 mating types. In paired cultures, antheridia were amphyginous, elongated, and very frequently bicellular. Oogonia showed a tapering base, and their outer wall was ornamented (bullate or verrucose), which is a distinctive character of very few *Phytophthora* species, including *P.* × *cambivora*. Oospores were globose, thick-walled, and plerotic, with a large central ooplast and an average diameter of 35 ± 4 µm. The proportion of A1 and A2 mating types was quite balanced among isolates from leaf baits (4 and 6 isolates out of 10, respectively) and soil (5 and 5 isolates out of 10, respectively), while the A2 type largely prevailed over the A1 type among isolates from roots (8 out of 10 isolates). 

Direct isolations from the bark of bleeding cankers yielded a *Phytophthora* species morphologically different from that recovered from the soil and roots. Beech trees with symptoms of bleeding stem cankers, five in all, were always near the shore of lakes or ponds and showed a high incidence of root rot. Cankers were on the main stem at the height of 60–120 cm from the soil, and bark pieces excised from the margin of active cankers were used for isolations. Eight randomly selected, single-hypha tip isolates obtained from these trees were characterized. Isolates of this *Phytophthora* species formed colonies with a rosette growth pattern on both V8A and PDA. They grew at 5 and 30 °C but did not grow at 35 °C. Optimum temperature for growth was at 25 °C. Chlamydospores were absent. Plugs from cultures grown on V8A flooded with non-sterile soil extract produced mycelium with globose hyphal swellings and non-papillate, obpyriform, internally and externally proliferating, and persistent sporangia. Sporangium dimensions averaged 50 ± 11 × 30 ± 5 μm. Isolates were self-sterile and did not form sexual structures when paired with tester strains of either the A1 or A2 *P. nicotianae* mating types.

**Figure 6:**
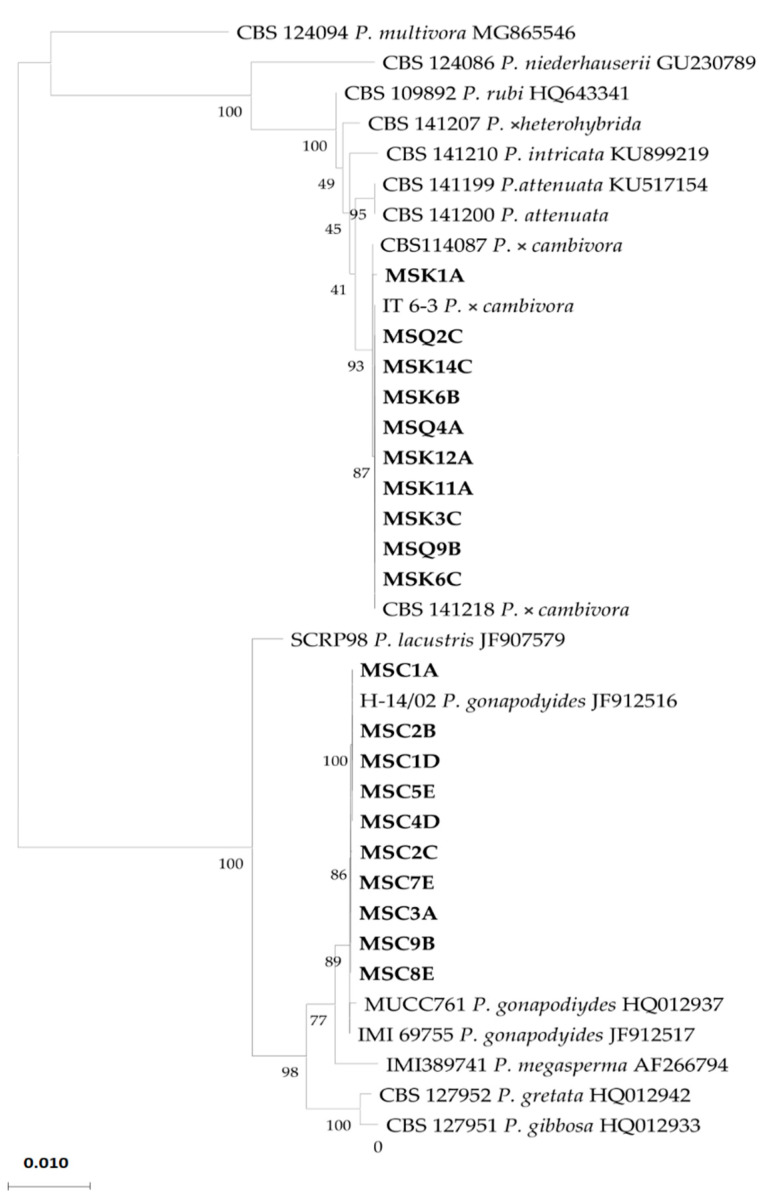
Internal transcribed spacer (ITS) and *Cox* I multilocus phylogenetic tree developed using the maximum likelihood method, based on the Tamura–Nei model. The tree with the greatest log likelihood (-5740.14) is shown. Relationships between the isolates from *Fagus sylvatica* sourced in southern Italy (in bold) and the CBS/IMI reference isolates of *Phytophthora gonapodyides*, *P. × cambivora*, and other *Phytophthora* spp. are shown.

Amplification and sequencing of the Internal Transcribed Spacer (ITS) regions of the ribosomal DNA (rDNA) of the isolates of the first species recovered from the beech trees revealed 99%100% identity with the sequence of the ex-neotype isolate of P. × *cambivora* (CBS141218), while isolates of the second species showed 100% identity with a reference isolate (IMI 69755) of *P. gonapodyides* [9]. In the tree obtained by the phylogenetic analysis of the combined data set of sequences from ITS and cytochrome c oxidase subunit I (*Cox* I) regions of isolates recovered from beech and sequences of *Phytophthora* species used as references, all isolates from the roots and soil clustered (bootstrap values of 1000 replicate) with the reference isolates of *P.* × *cambivora,* while isolates from the stem bark clustered with the reference isolates of *P. gonapodyides* (Figure 6). The accession numbers of the ITS and *Cox* I sequences of the isolates of *P.* × *cambivora* and *P. gonapodyides* sourced from the bark, roots, and rhizosphere soil of the beech trees in the Mount Soro forest stand deposited in GenBank are listed in Table 1.

There was a significant positive correlation (*p* ˂ 0.01) between the severity of beech tree decline and both the inoculum density of *P.* × *cambivora* in rhizosphere soil and the proportion of fine roots infected by this pathogen (Table 2). In general, *P. × cambivora* was recovered in 30 trees out of a total of 40 trees sampled, while *P. gonapodyides* was isolated in only 5 trees out of the total trees that were analyzed. Accordingly, when the 40 sampled beech trees were separated into two groups, i.e., severely declining trees and trees with mild symptoms of decline, the mean inoculum density and the proportion of infected fibrous roots were significantly higher in the former group. Mean values of inoculum density in the two groups of trees were 31.7 (range from 0 to 103) and 3.1 (range from 0 to 12) cfu/g of dry soil, respectively, while the mean proportions of infected fibrous roots were 48.6 (range from 2.5 to 88.75) and 16.1 (range from 0% to 66.5%), respectively. Moreover, the proportion of soil samples that were positive for *P.* × *cambivora* to the leaf-baiting test was significantly higher in severely declining trees than in healthy trees, 80% and 45%, respectively. Overall, in three soil samples, the presence of *P.* × *cambivora* was detected only with the leaf baiting, while in six samples, it was detected only with the dilution-plating assay. Eight samples, three from healthy trees and five from declining trees, resulted negative for *Phytophthora* with both assay methods. Not surprisingly, the mean proportion of fine roots infected by *P.* × *cambivora* in these eight samples was very low (6.25 ± 1.71%), even lower than the overall mean proportion of infected fine roots detected in healthy trees. The difference was significant according to the Student’s *t*-test at *p* < 0.01. *Phytophthora* × *cambivora* was always isolated from the fine roots and rhizosphere soil of the five trees showing stem bleeding cankers, from which *P. gonapodyides* was consistently recovered. The inoculum density of *P.* × *cambivora* in the rhizosphere soil of these five trees ranged from 45 to 84 cfus, while the proportion of fine roots infected by this pathogen ranged from 63% to 89%.

**Figure 7:**
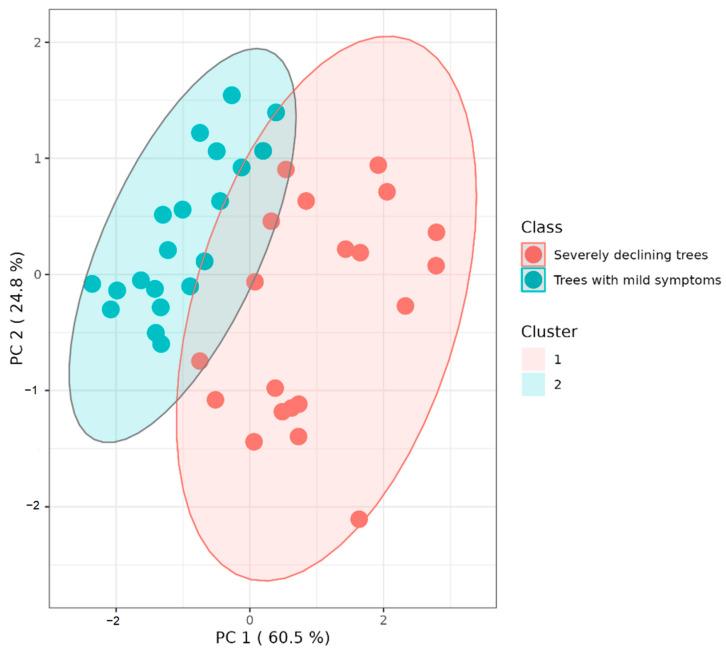
K-means cluster analysis of standardized values of three variables (proportion of beech roots infected by *Phytophtora* × *cambivora*, foliage transparency of beech trees, and inoculum density of *P.* × *cambivora* in rhizosphere soil of beech trees) grouping the samples into two clusters: trees with mild symptoms (blue dots) and severely declining trees (red dots).

K-means cluster analysis of standardized values of the three examined variables (proportion of beech roots infected by P. × cambivora, foliage transparency of beech trees, and inoculum density of P. × cambivora in rhizosphere soil of beech trees) grouped the samples into two clusters: trees with mild symptoms and severely declining trees. The sum of the principal component (PC) reached 85.3% of the total variance, of which PC1 (proportion of infected fibrous roots) represented 60.5% and PC2 (inoculum density) represented 24.8% of the total variances. In the area comprised within the two clusters, samples with symptoms intermediate to the two classes examined were aggregated (Figure 7), indicating that foliage transparency is a discriminant between the two classes.

## 4. Discussion

The present study, complementing a previous large-scale survey of PNAs in Sicily [11] and focusing on a single large beech stand on the slopes of Mount Soro, provided evidence that the oomycete *P.* × *cambivora*, in ITS *Phtytophthora* clade 7 [33], was widespread throughout the intensively surveyed stand, which confirms that this invasive oomycete has become established in the area of the Nebrodi RP. Moreover, the balanced presence of both mating types in the beech stand of Mount Soro is circumstantial evidence that *P.* × *cambivora* is reproducing sexually. Quite interestingly, a significant correlation came out between the health status of beech trees, as determined by visual inspection of the canopy, and both the amount of *P.* × *cambivora* inoculum in the rhizosphere soil of the beech trees and the percentage of the fine roots infected by this pathogen. K-means cluster analysis of the standardized values of the proportion of the beech roots infected by *Phytophthora* x *cambivora*, foliage transparency of the trees, and inoculum density of *P.* × *cambivora* in the rhizosphere soil, clearly discriminated beech trees with mild symptoms from severely declining trees. This strongly supports the hypothesis of a major role of *P.* × *cambivora* in the decline of beech stands in the Nebrodi RP [12]. More generally, it confirms that the destruction of the root system is a major driving factor of beech decline in sites where aggressive species of *Phytophthora* are present, provided that the environmental conditions are favorable to infections by these pathogens [10,34].

Numerous studies highlighted the association between the presence of soil-borne *Phytophthora* species and the decline of beech forests in Europe and the USA [3,4,5,6,7,8,9,10,11,35,36,37,38]. So far, 16 *Phytophthora* species, in addition to *P.* × *cambivora*, were recovered from beech forests across Europe. Most of them, including *P.* × *cambivora*, are considered pathogens of exotic origin, probably introduced because of human activities, while only a few species, such as *P. pseudosyringae*, *P. tyrrhenica*, and *P. vulcanica*, are supposed to be native to Europe [9,10,39,40,41]. Not all *Phytophthora* species recovered from beech stands in Europe are aggressive pathogens, and some species were found only sporadically [9,10]. *Phytophthora* × *cambivora* was the species most frequently associated with beech forest decline in Europe, which is amongst the most aggressive ones [10]; it has an almost worldwide distribution, is polyphagous, and can adapt to different environmental conditions. Moreover, Jung et al. [9] confirm the aggressiveness of *Phytophthora* × *cambivora* in pathogenicity tests on *Fagus sylvatica* seedlings, using *P*. x *cambivora* as a positive control. However, in a recent study aimed at investigating the association between the *Phytophthora* species and beech decline in Austria, *P.* × *cambivora* was exclusively recovered from geological substrates forming acidic soils rich in clay and sand [10], which is the same type of soil as that of the Mount Soro site. In beech stands in Austria and Bavaria (southern Germany), the altitudinal limits of *P.* × *cambivora* were 637 and 750 m a.s.l., respectively [10], while, at the Mount Soro site, this species was found up to an altitude of around 1800 m a.s.l., which is not surprising as the Nebrodi RP is located at the southernmost latitude of the geographical range of beech stands in Europe. In a previous survey of PNAs in Sicily, *P.* × *cambivora* was recovered from beech and oak stands at an altitude ranging from 660 to 1783 m a.s.l. [11]. Previous studies investigating the root status of declining trees in beech stands infested by *Phytophthora* species revealed a significant correlation between crown transparency and root health [10,42]. However, none of the previous studies related the amount of inoculum of *P.* *× cambivora* in the rhizosphere soil with both the severity of decline and root rot in mature beech trees. The present study is the first attempt to determine these relationships, with the aim of providing better insights into the role of *P.* *× cambivora* as the causative agent of beech decline in the Nebrodi RP.

The relationship between the level of *Phytophthora* population in the soil and Phytophthora root rot was thoroughly investigated in horticultural crops and ornamentals [43,44]. In a few cases, soil sampling protocols and threshold inoculum levels were defined, in order to forecast the risk of disease in relation to the susceptibility of the host-plant, to, thus, decide the interventions to prevent the disease or evaluate the effectiveness of disease management strategies [45,46]. The relative simplicity of agricultural systems facilitated the study of soil *Phytophthora* populations. These systems shared some fundamental characteristics, including the uniformity of the genotype and age of the plants, a low *Phytophthora* evenness, with the presence of a single or few species, and the virtual absence of perennial weeds. The presence of perennial weeds may interfere with the isolation, as some of them are potential hosts of other *Phytophthora* species or alternative hosts of the *Phytophthora* species associated preferentially with the tree crop. In this respect, the surveyed beech stand of Mount Soro could be compared with an agricultural system, as it was an almost pure beech stand, the beech trees were all of the same age, *P.* *× cambivora* was the sole or at least the largely prevalent *Phytophthora* species recovered from the soil, and the understory was almost absent due to the shadowing effect of trees in a very dense forest.

Quantitative studies of soil populations of *Phytophthora* were also performed in forest ecosystems to investigate the putative role of these oomycetes as a major contributing factor of the diebacks of white oak (*Quercus alba*) in the USA [47,48,49]. However, the results were, at least in part, contradictory. Declining white oak at sites infested by *P. cinnamomi* had significantly lower amounts of fine roots than white oak trees from noninfected sites, indicating a significant relationship between the presence of *P. cinnamomi* and white oak decline, while other *Phytophthora* species, including *P.* × *cambivora*, were recovered only occasionally [48]. When an intensive soil sampling was carried out, the ID of *P. cinnamomi* in the soil samples collected from declining white oak trees was significantly greater than the ID in soil samples from asymptomatic trees, so the authors hypothesized that this was the cause of the reduced number of fine roots observed in the declining trees [47,48]. However, when the sampling was scaled up and included a greater variety of sites within a wider geographical and climatic range, no constant association was found between the health status of tree crown, the presence of *P. cinnamomi* or other *Phytophthora* species, and their inoculum level in the soil, suggesting infections by *Phytophthora* species are not the only factor involved in the decline of white oak forests. Conversely, the health status of fine roots was related to the presence of *P. cinnamomi*, although only in sites located in hardiness zones five and six, as defined by the United States Department of Agriculture, suggesting climate plays a key role in the interaction between this oomycete and white oak trees [49]. Overall, from these results, the conclusion can be drawn that the decline of oak stands may be regarded as a complex disease sensu [50]. Several authors stressed the role of both local conditions and global warming in the triggering of beech and oak declines caused by *Phytophthora* species in Europe and the USA [3,4,10,12,42,51,52,53]. Others, while not excluding the role of environmental factors, assumed the mere presence in soil of invasive *Phytophthora* species, such as *P. cinnamomi, P.* × *cambivora, P. plurivora*, or *P. quercina*, is per se a potential health risk for oaks and beeches, due to their extreme susceptibility to Phytophthora root rot [10,54,55,56,57]. It is noteworthy that beech trees are less drought-tolerant than oaks and, consequently, are more vulnerable to the root damage caused by infections of the *Phytophthora* species [10].

*Phytophthora* × *cambivora* is the most common causal agent of bleeding stem cankers on beech trees in Europe. However, this symptom, associated sporadically with the decline of beech trees, may also be incited by other species of *Phytophthora* [10,36,37,58]. In the present study, only *P. gonapodyides*, in ITS *Phytophthora* clade 6, was recovered from the bark of bleeding stem cankers. This species is globally widespread and is mostly associated with aquatic or semi-aquatic habitats [59]. As a matter of fact, in Sicily, it occurs frequently in water courses and riparian vegetation [9,19]. Although it was reported as causal agent of bleeding stem cankers of beech in Sweden [60], it is generally regarded as an opportunistic pathogen. Consistently, in the present study, this *Phytophthora* species was isolated from the severely declining trees with a high percentage of their fine roots infected by the more aggressive *P.* × *cambivora*, on the banks of lakes or ponds subjected to temporarily flooding or prolonged soil waterlogging. A recent study aimed at evaluating the impact of infections by *P. cinnamomi* on the rhizosphere microbiome of avocado (*Persea americana*) showed Phytophthora root rot modifies the composition of the root-associated microbial community, favoring opportunistic fungal pathogens [61]. Extending the concept to beech decline, it may be inferred that root infections by *P.* × *cambivora* may predispose the beech trees to the infections of opportunistic pathogens, such as *P. gonapodyides*, or secondary invaders, which exacerbate the severity of decline but are not the primary pathogens. Therefore, in some cases the role of these fungi in the beech decline could be overestimated. By contrast, the role of *Phytophthora* species in complex diseases in general tends to be underestimated, as the isolation of these oomycetes on culture media commonly used for fungi is challenging, due to the poor competitive saprophytic ability of these pathogens.

## 5. Conclusions

This study showed that in a large beech stand in the Nebrodi RP, in Sicily, Italy, the severity of decline of trees, as determined by a visual inspection of the canopy, was related to the health status of the root system and the quantity of inoculum of *P.* × *cambivora* in the rhizosphere soil of trees, suggesting this oomycete has a primary role in determining the decline. This does not exclude other factors playing a significant role in the interaction between the host plant and the pathogen. The effect of global warming in triggering or favoring the beech decline across Europe has been hypothesized. However, the role of climate as the driving factor of the decline of beech trees in this area at the extreme southern edge of the geographical range of beech forests in Europe was out of the scope of this study. More research is needed to investigate the association between the site conditions and the incidence and severity of decline.

## Figures and Tables

**Table 1:** Isolates of *Phytophthora* sourced from rhizosphere soil and different tissues (fine roots and stem bark) of European beech (Fagus sylvatica) trees in the Nebrodi Regional Park of Sicily, Italy, as characterized in this study.

Isolate	*Phytophthora* Species	Source	Accession Numbers	
			ITS	*Cox* I
MSK1A	*Phytophthora* × *cambivora*	Soil and roots **^a^**	ON186623	ON227269
MSK6C	*P.*× *cambivora*	Soil and roots	ON186624	ON227270
MSQ9B	*P.* × *cambivora*	Soil and roots	ON186625	ON227271
MSK3C	*P.* × *cambivora*	Soil and roots	ON186626	ON227272
MSK11A	*P.* × *cambivora*	Soil and roots	ON186627	ON227273
MSK12A	*P.* × *cambivora*	Soil and roots	ON186628	ON227274
MSQ4A	*P.* × *cambivora*	Soil and roots	ON186629	ON227275
MSK6B	*P.* × *cambivora*	Soil and roots	ON186630	ON227276
MSK14C	*P.* × *cambivora*	Soil and roots	ON186631	ON227277
MSQ2C	*P.* × *cambivora*	Soil and roots	ON186632	ON227278
MSC1A	*P gonapodyides*	Stem bark	ON186633	ON227279
MSC2B	*P. gonapodyides*	Stem bark	ON186634	ON227280
MSC1D	*P. gonapodyides*	Stem bark	ON186635	ON227281
MSC5E	*P. gonapodyides*	Stem bark	ON186636	ON227282
MSC4D	*P. gonapodyides*	Stem bark	ON186637	ON227283
MSC2C	*P. gonapodyides*	Stem bark	ON186638	ON227284
MSC7E	*P. gonapodyides*	Stem bark	ON186639	ON227285
MSC3A	*P. gonapodyides*	Stem bark	ON186640	ON227286
MSC9B	*P. gonapodyides*	Stem bark	ON186641	ON227287
MSC8E	*P. gonapodyides*	Stem bark	ON186642	ON227288

^a^ Rhizosphere soil and fine roots.

**Table 2:** Pearson correlation coefficients between foliage transparency, as determined according to the classification of Schomacher et al. [17] and Eichhorn et al. [18], and inoculum density of *Phytophthora* × *cambivora*, in terms of cfu/g of soil and proportion (%) of fine roots infected by *P.* × *cambivora*, in 40 randomly selected beech trees.

	Foliage Transparency	Inoculum Density (cfu/g of Soil)	Proportion of Infected Fibrous Roots (%)
Proportion of infected fibrous roots (%)	0.466 **	0.508 **	1
Foliage transparency	1	0.548 **	0.466 **
Inoculum density (cfu/g of soil)	0.548 **	1	0.508 **

** Significant for *p* ˂ 0.01.

## Data Availability

Not applicable.
